# GPI-anchored single chain Fv - an effective way to capture transiently-exposed neutralization epitopes on HIV-1 envelope spike

**DOI:** 10.1186/1742-4690-7-79

**Published:** 2010-10-06

**Authors:** Michael Wen, Reetakshi Arora, Huiqiang Wang, Lihong Liu, Jason T Kimata, Paul Zhou

**Affiliations:** 1 The Unit of Anti-Viral Immunity and Genetic Therapy, the Key Laboratory of Molecular Virology and Immunology, the Institut Pasteur of Shanghai, Chinese Academy of Sciences, Shanghai, 200025, China; 2 Department of Molecular Virology and Microbiology, Baylor College of Medicine, Houston, Texas, 77030, USA

## Abstract

**Background:**

Identification of broad neutralization epitopes in HIV-1 envelope spikes is paramount for HIV-1 vaccine development. A few broad neutralization epitopes identified so far are present on the surface of native HIV-1 envelope spikes whose recognition by antibodies does not depend on conformational changes of the envelope spikes. However, HIV-1 envelope spikes also contain transiently-exposed neutralization epitopes, which are more difficult to identify.

**Results:**

In this study, we constructed single chain Fvs (scFvs) derived from seven human monoclonal antibodies and genetically linked them with or without a glycosyl-phosphatidylinositol (GPI) attachment signal. We show that with a GPI attachment signal the scFvs are targeted to lipid rafts of plasma membranes. In addition, we demonstrate that four of the GPI-anchored scFvs, but not their secreted counterparts, neutralize HIV-1 with various degrees of breadth and potency. Among them, GPI-anchored scFv (X5) exhibits extremely potent and broad neutralization activity against multiple clades of HIV-1 strains tested. Moreover, we show that GPI-anchored scFv (4E10) also exhibited more potent neutralization activity than its secretory counterpart. Finally, we demonstrate that expression of GPI-anchored scFv (X5) in the lipid raft of plasma membrane of human CD4^+ ^T cells confers long-term resistance to HIV-1 infection, HIV-1 envelope-mediated cell-cell fusion, and the infection of HIV-1 captured and transferred by human DCs.

**Conclusions:**

Thus GPI-anchored scFv could be used as a general and effective way to identify antibodies that react with transiently-exposed neutralization epitopes in envelope proteins of HIV-1 and other enveloped viruses. The GPI-anchored scFv (X5), because of its breadth and potency, should have a great potential to be developed into anti-viral agent for HIV-1 prevention and therapy.

## Background

Human Immunodeficiency Virus type 1 (HIV-1) envelope spike is a trimeric complex consisting of three non-covalently linked heterodimers of gp120 and gp41. Gp120, an exterior glycoprotein, mediates cell attachment, receptor and co-receptor binding. Gp41, a transmembrane glycoprotein, mediates viral and cell membrane fusion, which is critical for viral core to enter target cells. Both gp120 and gp41 are derived by cleavage of a common precursor gp160.

HIV-1 envelope spike also elicits antibody responses. Neutralizing antibodies block viral entry by recognizing epitopes on the envelope spike critical for its attachment, receptor and co-receptor interaction, or fusion and appear to be an important component of a protective immune response [1]. However, antibodies that can neutralize a broad range of primary HIV-1 isolates have been extremely difficult to generate [2]. Despite more than two decades of effort, only a few broadly neutralizing antibodies (2G12, b12, VRC001, VRC002, VRC003, PG9, PG16, 2F5 and 4E10/Z13) have been identified through screening antibody libraries or memory B cells from HIV-1 infected individuals [3-13]. Unfortunately, many efforts to elicit such antibody responses by active immunization have not been successful [14]. Interestingly, neutralization epitopes recognized by the aforementioned broadly neutralizing antibodies are present on the surface of the native spike and their recognition by the antibodies does not depend on conformational changes of envelope proteins.

Upon interaction with CD4 receptor, a lipid raft-associated protein [15-18], on the target cell surface, the native HIV-1 envelope spike goes through extensive conformational changes that allow additional binding to a co-receptor, CXCR4 for T-cell tropic strains or CCR5 for macrophage-tropic isolates. Co-receptor binding results in further conformational changes and leads to the insertion of the fusion peptide in gp41 into target cell membrane to drive the subsequent fusion event. During these conformational changes epitopes that are hidden from or not totally exposed on the surface of native spike are transiently exposed and become accessible to antibodies specific for these transiently-exposed epitopes. Likely, some of these epitopes are also neutralization epitopes. Based on this assumption, several groups reported using gp120-CD4 or gp120-CD4-CCR5 complex as immunogens to elicit antibodies that react with transiently-exposed neutralization epitopes or as selecting antigens for screening human phage display antibody libraries [19-21]. It was hypothesized that in these complexes HIV-1 envelope may stabilize some of the transiently-exposed epitopes so that antibodies present in the libraries that recognized these stabilized epitopes can be selected [22]. One notable example was the identification of a CD4-inducible antibody X5 in a phage display Fab antibody library with a gp120-CD4-CCR5 complex [21].

Previously, we unexpectedly found that by genetically linking the scFv of an anti-HIV-1 human antibody (TG15) to the transmembrane domain of subunit one of the type 1 interferon receptor, the cell-surface expressed scFv, but not its secretory form, we markedly inhibited HIV-1 entry and HIV-1 envelope-mediated cell-cell fusion [23,24]. The antibody recognizes the cluster II determinant (amino acid residues 644-663) which resides within the second heptad repeat (HR2) of HIV-1 gp41 [25]. HIV-1 gp41 mediated fusion is triggered by interaction between the second and the first heptad repeats, which converts a prehairpin gp41 trimer into a fusogenic three-hairpin bundle [26]. Similarly, it was reported that expressing a peptide derived from the HR2 domain on the surface of HIV-1-susceptible cells exhibits greater inhibitory effect on HIV-1 [27] and such an inhibition is achieved by capturing a gp41 fusion intermediate by the cell-surface expressed peptide prior to viral and cell membrane fusion [28]. Thus, it is clear that the cell-surface expressed scFv or peptide that recognizes or is derived from the HR2 domain can capture transiently-exposed epitopes in entry fusion intermediates. However, it is not clear whether transiently-exposed epitopes on HIV-1 envelope spikes other than that resides in the HR2 domain can also be captured by cell-surface expressed scFvs.

In nature, over 200 cell surface proteins with various functions are anchored to the plasma membrane by a covalently attached glycosyl-phosphatidylinositol (GPI) anchor [29]. Many GPI-anchored proteins are targeted into the lipid rafts of the plasma membrane. These specialized dynamic micro-domains are rich in cholesterol, sphingolipids and glycerophospholipids [30]. The lipid raft has been known to be a gateway for HIV-1 budding [31]. Furthermore, involvement of lipid rafts in HIV-1 entry into T cells and macrophages has also been proposed [15,31-33].

We therefore hypothesized that if one can express antibodies that react with transiently-exposed neutralization epitopes in a GPI anchored form and a GPI anchor can target these antibodies into the lipid rafts of plasma membranes of HIV-1-susceptible cells, these antibodies should neutralize infection. If correct, we predict that when the HIV-1 native spike interacts with the CD4 receptor, triggering a series of conformational changes, the transiently-exposed neutralization epitopes will be captured by GPI-anchored antibodies residing in the same lipid raft of the plasma membrane.

To test this hypothesis in this study, we constructed scFvs derived from seven different human monoclonal antibodies AB31, AB32, TG15, 4E10, 48d, X5 and AB65. AB65 recognizes the influenza hemagglutinin used here as negative control (Zhou, *et al. *data not shown). AB31 and AB32 are high affinity antibodies. AB31 recognizes cluster III determinant of gp41 and AB32 interacts with gp120, but its epitope is not well characterized [34]. Antibody (TG15) recognizes the cluster II determinant (amino acid residues 644-663) which resides within the second heptad repeat (HR2) of HIV-1 gp41 [23]. Antibodies 48d and X5 recognize distinct, but partially overlapped CD4 induced epitopes that are located close to both co-receptor-binding and CD4-binding sites of gp120 [21,35,36]. Antibody 4E10 that recognizes a linear epitope residing in the membrane proximate region of gp41 is a neutralizing antibody [7]. Here, we show that by genetically linking the scFvs with a GPI attachment signal derived from decay accelerating factor (DAF) [37] the scFvs are targeted to lipid rafts of plasma membranes. In addition, we demonstrate that the four of these GPI-anchored scFvs (X5, 48d, AB32 and TG15), but not their secretory counterparts, neutralize HIV-1 with various degrees of breadth and potency. Among them, GPI-anchored scFv (X5) exhibits extremely potent and broad neutralization activity against multiple clades of HIV-1 strains tested. Moreover, we show that GPI-anchored scFv (4E10) also exhibited more potent neutralization activity than its secretory counterpart. Finally, we demonstrate that expression of GPI-anchored scFv (X5) in the lipid raft of plasma membrane of human CD4^+ ^T cells confers long-term resistance to HIV-1 infection, HIV-1 envelope-mediated cell-cell fusion and the infection of HIV-1 captured and transferred by human DCs. Thus, we conclude that GPI-anchored scFv is an effective way to capture transiently-exposed neutralization epitopes in the HIV-1 envelope spike.

## Results

### Expression of scFv in the lipid raft of plasma membrane through a GPI anchor

**Figure 1:**
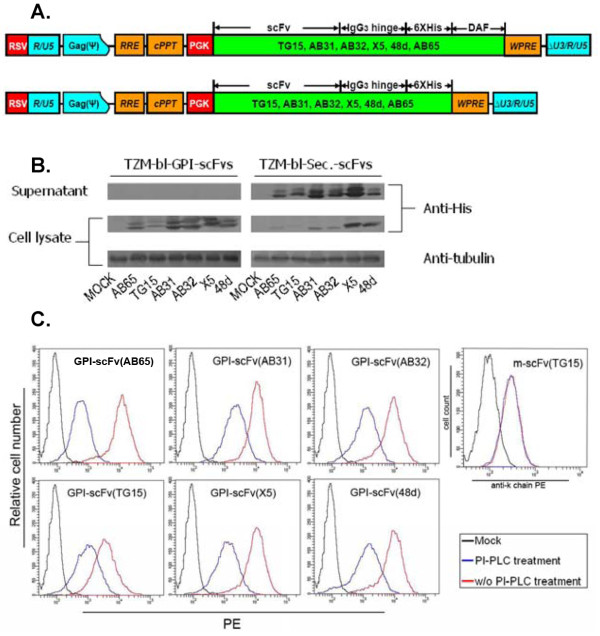
**Expression of secretory and GPI-anchored scFvs in transduced TZM.bl cells**. ***A***. Schematic diagram of the lentiviral vectors pRRL-scFv/hinge/his-tag/DAF and pRRL-scFv/hinge/his-tag. Single chain Fvs (scFvs) were derived from seven human monoclonal antibodies AB31, AB32, TG15, 48d, X5 and AB65; hinge: a human IgG3 hinge region; his-tag: a 6 histidine residue tag; DAF: the C-terminal 34 amino acid residues of decay accelerating factor. ***B***. Western blot analysis of expression of scFvs (AB31, AB32, TG15, 48d, X5 and AB65) in TZM.bl cells transduced with lentiviral vectors pRRL-scFv/hinge/his-tag/DAF and pRRL-scFv/hinge/his-tag. GPI-scFv: GPI-anchored scFv; Sec-scFv: secretory scFv; anti-his: anti-his-tag antibody. ***C***. FACS analysis of cell surface expression of scFv/hinge/histag/DAF in mock-, scFvs (AB31, AB32, TG15, 48d, X5 and AB65)/hinge/histag/DAF- or m-scFv(TG15)-transduced TZM.bl cells with or without PI-PLC treatment.

To generate GPI-anchored and secretory scFvs, the sequences encoding scFvs derived from seven different human antibodies AB31, AB32, TG15, 4E10, 48d, X5 and AB65 were genetically linked with the sequence encoding a his-tagged IgG3 hinge region and with or without the sequence encoding a GPI attachment signal of DAF [37]. The fusion genes scFv/IgG3 hinge/his-tag/DAF and scFv/IgG3 hinge/his-tag were inserted into a third generation lentiviral vector pRRL (Figure 1A). The recombinant viruses were then generated as described before [38] and used to transduce TZM.bl cells and human CD4^+ ^T cells CEMss and CEMss-CCR5 (see below). The expression of transgenes and localization of transgene products in the transduced cells were carefully studied.

Figure 1B shows the expression of scFvs/hinge/his-tag/DAF and scFvs/hinge/his-tag in cell lysates and culture supernatants of transduced TZM.bl cells by western blot using anti-his-tag and anti-tubulin antibodies. As expected, without a GPI attachment signal, all scFvs were detected in both culture supernatants and cell lysates with a majority in supernatants (the right panel). By contrast, all scFvs with a GPI attachment signal were only detected in cell lysates, but not in culture supernatants (the left panel). These data indicate that inclusion of a GPI attachment signal prevents secretion of the scFvs.

To determine if scFvs/hinge/his-tag/DAF were expressed on the cell surface through a GPI anchor, scFv/hinge/his-tag/DAF-transduced TZM.bl cells were treated with or without phosphatidylinositol-specific phospholipase C (PI-PLC) and stained with anti-his-tag antibody followed by FACS analysis. As a control, cells transduced with previously reported m-scFv (TG15), a cell-surface expressed scFv (TG15) with a conventional transmembrane domain [23] went through the same PI-PLC treatment and staining processes. Figure 1C shows that all scFv/hinge/his-tag/DAFs express highly on cell surface (about 10-fold higher than that of m-scFv) and the expression were substantially reduced with PI-PLC treatment. In contrast, no reduction in cell surface expression of scFv was observed in m-scFv-transduced cells, indicating that the expression of scFv/hinge/his-tag/DAF on the cell surface is indeed through a GPI anchor. In addition, cell surface expression of GPI-anchored scFv (4E10) along with GPI-anchored scFvs (AB65 and X5) was also analyzed by immune staining and FACS analysis. Additional File 1 shows that cell surface expression of GPI-anchored scFv (4E10) is similar to those GPI-anchored scFvs (AB65 and X5). Thus, for the sake of simplicity in the remaining text we will refer the scFv/hinge/his-tag/DAF as GPI-scFv and scFv/hinge/his-tag as secretory scFv.

**Figure 2:**
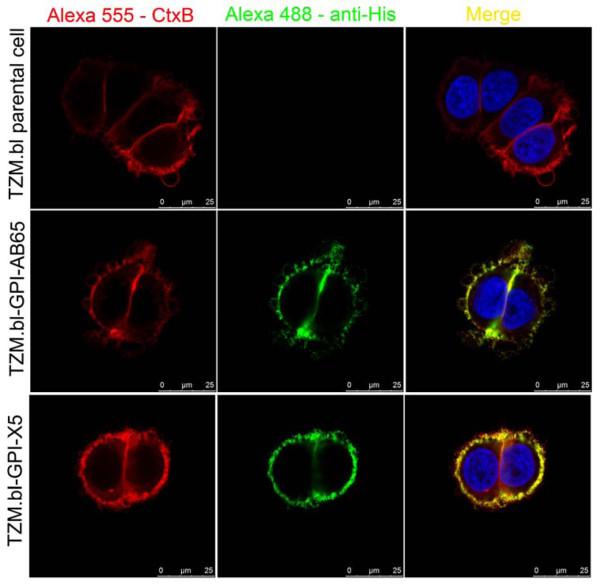
**Localization of GPI-anchored scFvs in transduced TZM.bl cells**. Confocal analysis of mock- or GPI-scFvs (AB65 and X5)-transduced TZM.bl cells. CtxB: cells were stained with Alexa 555-conjugated cholera toxin B subunit; anti-his: cells were stained with mouse anti-his-tag antibody followed by Alexa 488-conjugated goat anti-mouse IgG antibody.

To determine if GPI-scFvs are located in the lipid rafts of plasma membranes, mock- and GPI-scFv (AB65 and X5)-transduced TZM.bl cells were seeded into wells of cover slip chambers and cultured overnight. Cells were then fixed with 4% formaldehyde and co-stained with 1) mouse anti-his-tag antibody followed by Alexa 488-conjugated goat anti-mouse IgG antibody; 2) Alexa 555-conjugated cholera toxin subunit B (CtxB); and 3) DAPI. CtxB interacts with GM1 (a lipid raft marker) on the cell surface. Figure 2 shows that both GPI-scFvs (AB65 and X5) are co-localized with GM1 on cell surface, implying that they are located in the lipid raft of the plasma membrane.

### GPI-scFv (X5) exhibits remarkable degree of breadth and potency against HIV-1

Next, we compared CD4, CCR5 and CXCR4 expression in the secretory and GPI-scFv-transduced TZM.bl cells and found that there is no significant difference in their expression compared to mock-transduced TZM.bl cells, suggesting that the expression of transgenes does not alter the expression of the receptor and the coreceptors for HIV-1 in the transduced cells (Additional File 2). Neither did we find that the expression of the transgenes alters the cell growth (Zhou *et al*. data not shown).

**Figure 3:**
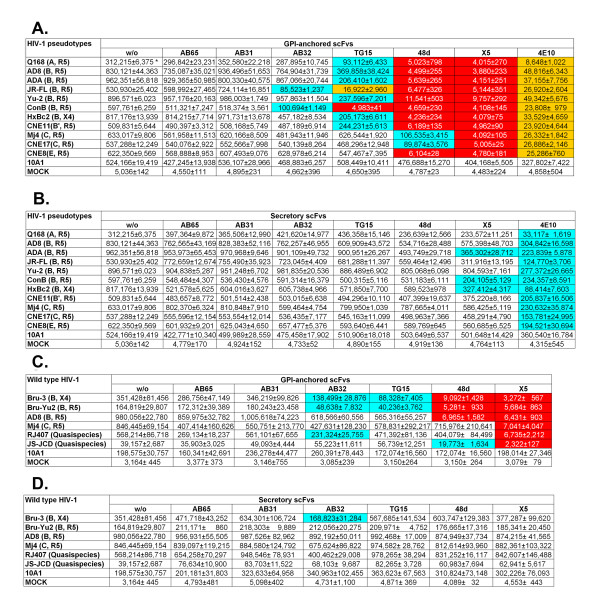
**Effect of secretory and GPI scFvs (AB31, AB32, TG15, 4E10, 48d, X5 and AB65) on infection of HIV-1 viruses and pseudotypes**. ***A. ***Effect of GPI-scFvs on transduction efficiency of HIV-1 and 10A1 pseudotypes into GPI-scFv-transduced TZM.bl. w/o: parental TZM.bl cells; *mean and standard deviation of relative luciferase activity. ***B. ***Effect of secretory scFvs on transduction efficiency of HIV-1 and 10A1 pseudotypes into secretory scFv-transduced TZM.bl. w/o: parental TZM.bl cells. ***C. ***Effect of GPI-scFvs on wild type HIV-1 infection in GPI-scFv-transduced TZM.bl. w/o: parental TZM.bl cells. ***D. ***Effect of secretory scFvs on wild type HIV-1 infection in secretory scFv-transduced TZM.bl. w/o: parental TZM.bl cells. Blue color-coated: > or = 50% inhibition; Orange color-coated: > or = 90% inhibition; Red color-coated: >or = 99% inhibition. The percentage of inhibition was based on the following calculation: (RLA in virus alone to a given transduced cell - RLA in no virus to the same transduced cell)/(RLA in virus alone to parental cells - RLA in no virus to parental cell).

To test neutralization activity of the secretory versus the GPI-scFvs against HIV-1, an eleven multiclade HIV-1 pseudotype panel and a retroviral envelope 10A1 pseudotype were used to infect transduced TZM.bl cells in a single-round infection experiment [23]. The retroviral envelope 10A1 recognizes either Ram-1 or Glvr-1 as a receptor for cell entry [39] and used here as negative control. The eleven HIV-1 pseudotypes consist of HIV-1 envelopes derived from clade A (Q168), clade B (HxBc2, JF-RL, ADA, AD8, Yu2 and consensus B), clade B' (CNE11), clade C (Mj4 and CNE17) and clade E (CNE8). Figure 3 shows mean and standard deviation of relative luciferase activity (RLA) in mock-, secretory and GPI-scFv-transduced cells infected with these pseudotypes. Compared to mock-transduced cells, cells transduced with all secretory and GPI-anchored scFvs did not show significant neutralization activity against 10A1 pseudotypes control (Figure 3A and 3B). Compared to mock-transduced cells, cells transduced with secretory scFvs (AB65, AB31, AB32, TG15, and 48d) did not show significant neutralization activity against any of these HIV-1 pseudotypes tested. Cells transduced with secretory scFv (X5) showed low degree of neutralization activity against 3 of 11 HIV-1 pseudotypes (ADA, Consensus B and Mj4). In contrast, cells transduced with secretory scFv (4E10) exhibited more than 50% neutralization activity against all 11 HIV-1 pseudotypes tested (Figure 3B). Compared to mock-transduced cells, cells transduced with GPI-scFvs show various degree of potency and breadth against HIV-1 pseudotypes (Figure 3A). Like cells transduced with GPI-scFvs (AB65) control, cells transduced with GPI-scFvs (AB31) did not show neutralization breadth and potency against any of these pseudotypes tested. Cells transduced with GPI-scFv (AB32) neutralized 2 of 11 HIV-1 pseudotypes (JR-FL and Consensus B) with low degree of potency. Cells transduced with GPI-scFv (TG15) neutralized 8 of 11 HIV-1 pseudoviruses expressing envelopes derived from clades A, B and B' with various degree of potency, but not clades C and E. Cells transduced with GPI-scFv (4E10) neutralized all 11 HIV-1 pseudotypes with increased potency (more than 90% neutralization activity) as compared to cells transduced with secretory scFv (4E10). Cells transduced with GPI-scFvs (48d) neutralized all 11 HIV-1 pseudotypes with great degree of potency against HIV-1 pseudotypes expressing envelopes derived from clades A, B, B' and E, but less potent against envelope derived from clade C. Strikingly, cells transduced with GPI-scFv (X5) neutralized all 11 HIV-1 pseudotypes with remarkable degree of potency.

We next tested neutralization activity of the secretory versus the GPI-scFvs against 6 replication competent HIV-1 strains including two clinical isolates (quasispecies). Figure 3C and 3D show mean and standard deviation of RLA in mock-, secretory and GPI-scFv-transduced cells infected with these HIV-1 strains. Compared to mock-transduced cells, cells transduced with all secretory scFvs did not show significant neutralization activity against any of these HIV-1 strains tested (Figure 3D). In contrast, cells transduced with the GPI-scFvs show various degree of breadth and potency (Figure 3C). Like cells transduced with GPI-scFv (AB65) control, cells transduced with GPI-scFv (AB31) did not neutralize any of these HIV-1 strains tested. Cells transduced with GPI-scFv (AB32 and TG15) neutralized 2 HIV-1 strains (Bru-3, and Bru-Yu2) with a low degree of potency; and cells transduced with GPI-scFv (48d) neutralized 4 viruses including one clinical quasispecies (Bru-3, Bru-Yu2, AD8 and JS-JCD) with various degree of potency. Interestingly, cells transduced with GPI-scFv (X5) neutralized all 6 viruses with a remarkable degree of potency.

### Potent inhibition of HIV-1 by GPI-scFv (X5) does not require additional sCD4

It was previously showed that the scFv (X5) neutralizes HIV-1 better than the Fab and the whole IgG [40] and the binding and neutralizing capability of scFv (X5) can be greatly enhanced by adding soluble extracellular domains of human CD4 (sCD4) [21,41,42]. We therefore produced and purified soluble CD4 using the drosophila S2 expression system (see Additional File 3). We then tested the effect of sCD4 doses on HIV-1 infection (Bru-3, Bru-Yu2 and Mj4) and found that at 1 μg/ml or higher a concentration-dependent inhibition of HIV-1 infection by sCD4 was observed; while below 1 μg/ml no significant inhibition by sCD4 was observed (Zhou et al. data not shown). Thus, we chose sCD4 at the concentration of 0.3 μg/ml in the subsequent post-CD4 experiments as described before [43].

**Figure 4:**
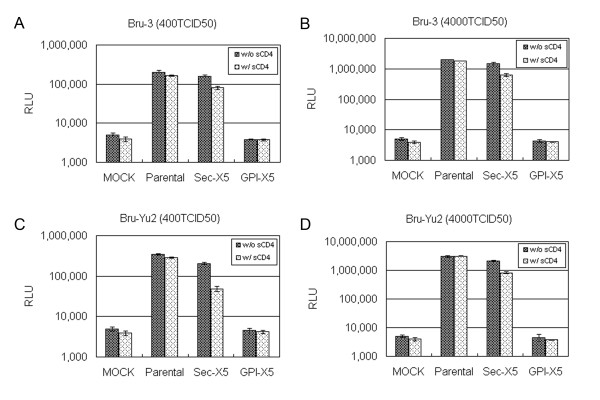
**Potent inhibition of HIV-1 by GPI-scFv (X5) does not require additional sCD4**. sCD4: soluble, extracellular domains of human CD4; w/o or w/sCD4: with or without pre-incubation of viruses with sCD4; y-axis: mean and standard deviation of relative luciferase activity; mock: negative control, background relative luciferase activity in uninfected TZM.bl cells. ***A. ***Cells infected with 400 TCID_50 _of HIV-1 Bru-3 with or without pre-incubation with sCD4; ***B. ***Cells infected with 4,000 TCID_50 _of HIV-1 Bru-3 with or without pre-incubation with sCD4; ***C. ***Cells infected with 400 TCID_50 _of HIV-1 Bru-Yu2 with or without pre-incubation with sCD4; ***D. ***Cells infected with 4,000 TCID_50 _of HIV-1 Bru-Yu2 with or without pre-incubation with sCD4.

Figure 4 shows mean and standard deviation of RLA in mock-, secretory and GPI-scFv-transduced TZM.bl cells infected with or without HIV-1 Bru-3 or Bru-Yu2 that were pre-incubated with or without sCD4. Pre-incubation of 400 and 4,000 TCID_50 _of these two HIV-1 strains with sCD4 greatly enhances inhibition in cells transduced with secretory scFv (X5); while complete inhibition was observed in GPI-scFv (X5)-transduced cells infected with 400 and 4,000 TCID_50 _of these two HIV-1 strains, regardless whether the viruses were pre-incubated with sCD4 or not (Figure 4A-D). Thus, these results clearly show that while sCD4 enhances inhibition by secretory scFv (X5); GPI-scFv (X5) exhibits the greatest potency of inhibition, which is totally independent of addition of sCD4.

### GPI-scFv (X5) confers long-term resistance to HIV-1 in human CD4^+ ^T cells

**Figure 5:**
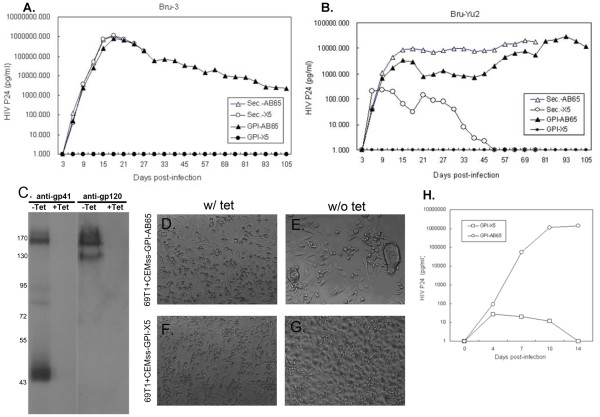
**Effect of GPI-scFv (X5) on anti-HIV-1 activity of transduced human CD4^+ ^T cells**. ***A. ***GPI-scFv (X5) confers long-term resistance to HIV-1 Bru-3 in human CD4^+ ^T cells. sec-AB65: CEMss-CCR5 cells transduced with secretory scFv (AB65); sec-X5: CEMss-CCR5 cells transduced with secretory scFv (X5); GPI-AB65: CEMss-CCR5 cells transduced with GPI-scFv (AB65); GPI-X5: CEMss-CCR5 cells transduced with GPI-scFv (X5). ***B. ***GPI-scFv (X5) confers long-term resistance to HIV-1 Bru-Yu2 in human CD4^+ ^T cells. ***C. ***Western blot analysis of HIV-1 gp160, gp120 and gp41 expression by anti-HIV-1 gp120 and gp41 antibodies in 69 T1RevEnv cells with or without treatment of tetracycline. Lane 1: 69 T1RevEnv cells without treatment of tetracycline stained with anti-HIV-1 gp41 antibody; lane 2: 69TiRevEnv cells with treatment of tetracycline stained with anti-HIV-1 gp41 antibody; lane 3: 69TiRevEnv cells without treatment of tetracycline stained with anti-HIV-1 gp120 antibody; lane 4: 69TiRevEnv cells with treatment of tetracycline stained with anti-HIV-1 gp120 antibody. ***D. ***Cell morphology 20 hours after coculturing tetracycline-treated 69TiRevEnv cells with CEMss-CCR5-GPI-scFv (AB65). ***E. ***Cell morphology 20 hours after coculturing tetracycline-untreated 69TiRevEnv cells with CEMss-CCR5-GPI-scFv (AB65). ***F. ***Cell morphology 20 hours after coculturing tetracycline-treated 69TiRevEnv cells with CEMss-CCR5-GPI-scFv (X5). ***G. ***Cell morphology 20 hours after coculturing tetracycline-untreated 69TiRevEnv cells with CEMss-CCR5-GPI-scFv (X5). ***H. ***GPI-scFv (X5) blocks the infection of HIV-1captured and transferred by human DCs. GPI-X5: co-culturing infected human DC with CEMss cells transduced with GPI-scFv (X5); GPI-AB65: co-culturing infected human DC with CEMss cells-transduced with GPI-scFv (AB65).

Next, we evaluated if GPI-scFv (X5) would confer the long-term resistance to HIV-1 in human CD4^+ ^T cells. Human CD4^+ ^cell line CEMss was first transfected with a retroviral vector expressing human CCR5. After stable CEMss-CCR5 cells were established, they were further transduced with secretory and GPI-scFv (X5 and AB65). The expression of secretory and GPI-scFvs as well as CD4, CCR5 and CXCR4 in transduced CEMss-CCR5 cells were tested by western blot and immune staining followed by FACS analysis as described above (see Additional File 4). Transduced CEMss-CCR5 cells were then infected with HIV-1 strains Bru-3 and Bru-Yu2 at multiple of infection of 0.01 as described before [23] and cultured in the complete DMEM medium for 75 to 105 days, except for cells transduced with secretory scFvs (AB65 and X5) and infected with Bru-3 (the culture of these cells was terminated on day 27 post infection). As shown in Figure 5A and 5B, replication of both HIV-1 Bru-3 and Bru-Yu2 was completely inhibited in cells transduced with GPI-scFv (X5) throughout the experiments. In contrast, robust replication of HIV-1 Bru-3 and Bru-Yu2 was observed in cells transduced with secretory scFv (AB65) and GPI-scFv (AB65) controls. For cells transduced with secretory scFv (X5) and infected with HIV-1 Bru-3, HIV-1 replication was as robust as secretory scFv (AB65) and GPI-scFv (AB65) controls. By contrast, for cells transduced with secretory scFv (X5) and infected with HIV-1 Bru-Yu2, robust HIV-1 replication was observed in the first 6 days and then slowly dropped to the undetectable level on day 51 and thereafter. These data demonstrated that GPI-scFv (X5) completely inhibits the infection of HIV-1 Bru-3 and Bru-Yu2. By so doing it maintains long-term resistance to HIV-1. On the contrary, secretory scFv (X5) cannot inhibit the infection and replication of fast replicating HIV-1 like Bru-3, but can partially inhibits the replication of relatively slow replicating HIV-1 like Bru-Yu2.

### GPI-scFv (X5) blocks HIV-1 envelope-mediated cell-cell fusion

To evaluate the effect of GPI-scFv (X5) on HIV-1 envelope-mediated cell-cell fusion, the GPI-scFv (X5 and AB65)-transduced CEMss-CCR5 cells were co-cultured with 69TiRevEnv cells as previously described [44]. The latter contains a HIV-1 envelope gene (pLAI3) under a Tet-off promoter. In the presence of tetracycline, binding of tetracycline to Tet transactivator (tTA) causes conformational change of tTA, which blocks tTA binding to the Tet-off promoter and prevents HIV-1 envelope protein expression; in the absence of tetracycline, tTA binds to and transactivates the Tet-off promoter resulting in HIV-1 envelope protein expression (Figure 5C). Co-culturing GPI-scFv (AB65 and X5)-transduced CEMss-CCR5 cells with tetracycline-treated 69TiRevEnv cells results in no cell-cell fusion (Figure 5D and 5E). In contrast, co-culturing GPI-scFv (AB65)-transduced CEMss-CCR5 cells with tetracycline-untreated 69TiRevEnv cells results in massive cell-cell fusion (Figure 5F). The fusion begins after 6 hours and peaks at 20 hours. Importantly, no cell-cell fusion was observed after 20 hour's co-culturing GPI-scFv (X5)-transduced CEMss-CCR5 cells with tetracycline-untreated 69TiRevEnv cells (Figure 5G). The experiment was repeated twice with similar results. Thus, these data demonstrated that GPI-scFv (X5) completely inhibits HIV-1 envelope-mediated cell-cell fusion.

### GPI-scFv (X5) blocks the infection of HIV-1captured and transferred by human DCs

To test the effect of GPI-scFv (X5) on the infection of HIV-1 captured and transferred by human DCs, monocyte-derived human DCs were incubated with HIV-1 NL4-3. Cells were then washed extensively to remove free viruses. Infected DCs were then co-cultured with GPI-scFv (X5 and AB65) transduced CEMss cells for 14 days. HIV-1 replication was measured by HIV-1 p24 assay as described above. As shown Figure 5H, co-culturing GPI-scFv (AB65)-transduced CEMss cells with HIV-1 infected monocyte-derived human DCs results in high p24 expression, indicating robust replication of HIV-1. In contrast, co-culturing GPI-scFv (X5)-transduced CEMss cells with HIV-1 infected monocyte-derived human DCs results in very low level of HIV-1 p24 during the first 4 days and drops off thereafter, indicating inhibition of viral replication. This low level of HIV-1 replication detected in the coculture of GPI-scFv (X5)-transduced CEMss cells and HIV-1 infected monocyte-derived human DCs likely reflects slow and covert HIV-1 replication in monocyte-derived human DCs as previously reported [45]. Thus, the data clearly demonstrated that GPI-scFv (X5) can neutralize HIV-1 captured and transferred by human DCs.

### GPI-scFv (X5) does not inhibit transduction by VSV G pseudotyped HIV-1 vector

**Figure 6:**
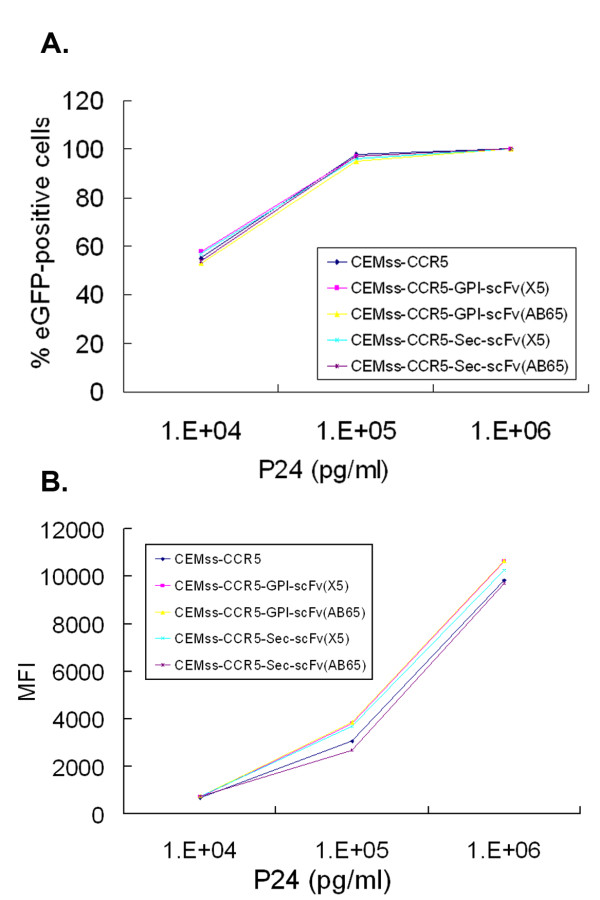
**eGFP expression in parental CEMss-CCR5 cells and CEMss-CCR5 expressing GPI-scFvs (AB65 and X5) and secretory scFvs (AB65 and X5) transduced with VSV-G pseudotyped HIV-1 vector**. ***A. ***% of eGFP positive cells; ***B. ***MFI.

Finally we transduced parental CEMss-CCR5 cells and CEMss-CCR5 [GPI-scFvs (AB65 and X5) secretory scFvs (AB65 and X5)] with VSV-G pseudotyped HIV-1 vector expressing enhanced green fluorescent protein (eGFP) as described before [23]. Because VSV G envelope interacts with lipid moiety in the lipid bilayer of the plasmic membrane, vectors by pass the requirement of the interaction between HIV-1 envelope and its receptor and co-receptor to enter cells. We found that in all three doses tested, transducing parental CEMss-CCR5 cells and CEMss-CCR5 [GPI-scFvs (AB65 and X5) secretory scFvs (AB65 and X5)] with VSV-G pseudotypes results in similar vector dose-dependent transduction efficiency and transgene expression (Figure 6). These results demonstrate that the GPI-scFv (X5) does not inhibit the VSV G envelope-mediated viral entry, reverse transcription, integration, or postintegration protein expression of HIV-1 vector, indicating that the potent inhibition of HIV-1 replication and HIV-1 envelope-mediated cell-cell fusion seen in the GPI-scFv (X5)-transduced CEMss cells (Figure 5) is HIV-1 envelope-specific and at the level of viral entry.

## Discussion

In this study we demonstrate that by genetically linking scFvs with GPI-attachment signal scFvs are expressed in the lipid raft of plasma membrane through a GPI anchor (Figure 1 and 2). GPI-scFvs, but not secretory scFvs, of the antibodies (AB32, TG15, 48d and X5) that recognize transiently-exposed epitopes on HIV-1 envelope spike neutralize HIV-1 with various degrees of breadth and potency. Among them, GPI-scFv (X5) exhibits extremely potent and broad neutralization activity against multiple clades of HIV-1 (Figures 3). Moreover, we show that GPI-anchored scFv (4E10) also exhibited more potent neutralization activity than its secretory counterpart (Figures 3). Importantly, the expression of GPI-scFv (X5) on the surface of human CD4^+ ^T cells confers long-term resistance to HIV-1 infection, HIV-1 envelope-mediated cell-cell fusion and the infection of HIV-1 captured and transferred by human DCs (Figure 5). Thus, targeting scFv of antibody molecules in the lipid rafts of plasma membranes of HIV-1 susceptible cells through a GPI anchor is an effective way to capture transiently exposed neutralization epitopes of HIV-1 envelope spike. GPI-scFv (X5) with such remarkable breadth and potency should have a potential to be developed into an anti-viral agent for HIV-1 prevention and therapy. For example, similar to those recently reported by DiGiusto et al. [46], GPI-scFv (X5) could be delivered into hematopoietic progenitor cells of HIV-1 patients *ex vivo *through lentiviral vector and transduced cells could then be transfused to the patients. However, in order to achieve clinical efficacy with this gene therapy approach, many hurdles, such as low degree of transduction efficiency and engraftment, difficulty in maintenance of self renewal as well as hematopoietic linage cell differentiation of transduced hematopoietic progenitor cells, unsustainable transgene expression, potential insertion mutagenesis, have to be overcome.

Interestingly, although their level of expression on the cell surface is similar (Figure 1C), 6 anti-HIV-1 gp120 or gp41 GPI-scFvs tested in this study display the wide spectrum of breadth and potency against 11 HIV-1 pseudotypes and 6 wild type HIV-1 strains tested (Figure 3). At one end of the spectrum, GPI-scFv (AB31) does not show significant neutralization activity against any of these HIV-1 viruses; while on the other end of the spectrum, GPI-scFv (X5) neutralizes all HIV-1 viruses with remarkable degree of potency. Between these two extremes, GPI-scFvs (AB32, TG15, 4E10 and 48d) show a progressive increase of breadth and potency. Furthermore, although they both recognize CD4-induced epitopes, GPI-scFv (48d) and GPI-scFv (X5) show different potency against HIV-1 strains from clade C, but similar potency against HIV-1 strains from clades A, B, B' and E. Thus these results strongly suggest that the breadth and potency of a given GPI-scFv does not just depend on local concentration, but it is dependent on the fine specificity of the transiently-exposed neutralization epitope it targets.

In this study, we also show that potent inhibition of HIV-1 by GPI-scFv (X5) does not depend on additional sCD4 (see Figure 4). We believe that this likely has something to do with the X5 epitope. The X5 epitope resides within a conserved region (amino acid residues 417 to 434) of the gp120 core, particularly in the vicinity of amino acid residues at positions 423 and 432, which is in the proximity of the CD4 and coreceptor binding sites [35]. According to the crystal structure of gp120 core with or without sCD4 [41,42] as well as crystal structure of a complex containing V3-containing gp120 core, the CD4 receptor and the Fab (X5) [47], the amino acid residues 417 to 434 reside in β19, β20 and β21 of the gp120 core. The β20 and the β21 form an anti-parallel β sheet. Without adding sCD4, β19 and β20/β21 are away from each other, the β19 is covered by V3 loop and the β20/β21 is partially covered by V1/V2 loop. Because of this, secretory scFv (X5) does not have neutralization activity. In contrast, adding sCD4 induces a CD4-induced conformation of gp120. In this conformation, the β19 and the β20/β21 are not only totally exposed, but also become close to each other [42]. As a result, adding sCD4 greatly enhances neutralization activity of secretory scFv (X5) (Figure 4). However, it is very plausible that even in the presence of sCD4 CD4-induced conformation of the HIV-1 spike is transient, so that secretory scFv (X5) may not be able to catch all X5 epitopes on the spikes of the virion. As a result, neutralization activity is not optimal. GPI-scFv (X5), on the other hand, due to its lipid raft location and local concentration, may have much better access to its transiently-exposed neutralization epitope upon interaction of CD4 with the native envelope spike. Here CD4, a raft-associated protein, resides in the lipid raft of the plasma membrane of target cells, which is the same location as GPI-scFv (X5). As a result, GPI-scFv (X5) may quickly and efficiently capture the CD4-induced neutralization epitope and completely block the envelope spike in a pre-fusion intermediate step. However, the underlying mechanism of great potency of GPI-scFv (X5) remains to be elucidated.

There are at least two implications of the findings in this study. First, given the promising results obtained in this study, it is plausible to develop GPI-scFvs into a standard approach for identifying antibodies that recognize transiently-exposed epitopes on HIV-1 envelope spike. It is conceivable that for many anti-HIV-1 gp120 and gp41 antibodies that have no or poor neutralization activity against native envelope spikes, GPI-scFvs of these antibodies can be made and tested against a panel of different HIV-1 variants and subtypes. If some of these GPI-scFvs exhibit good neutralization activity, one can then use these GPI-scFvs in combination with epitope mapping methods (such as alanine-scanning mutants) to identify transiently-exposed neutralization epitopes in the HIV-1 envelope spike. Second, it is also plausible that GPI-scFvs be used to identify antibodies that recognize transiently-exposed epitopes and then to identify transiently-exposed neutralization epitopes of other viruses, particularly of those enveloped viruses whose entry into target cells depend on lipid raft and the interaction of those envelope glycoprotein with receptor results in conformational changes. It has been shown that glycoproteins of influenza virus, murine leukemia virus, measles virus and Ebola virus are associated with the lipid raft of plasma membrane [48-52]. The entry of many enveloped viruses, such as vaccinia virus [53], severe acute respiratory syndrome-coronavirus [54], duck hepatitis B virus [55], murine leukemia virus [56] and herpes simplex virus [57], requires cholesterol in the target cell membrane. Therefore, development of GPI-scFvs that are derived from antibodies that recognize transiently-exposed epitopes in glycoproteins of these viruses may also be an effective way to inhibit cell entry of these viruses.

## Conclusions

GPI anchored scFvs that recognize different transiently-exposed epitopes on HIV-1 envelope spike exhibit various degrees of breadth and potency. Among them, GPI-scFv (X5) that recognizes the epitope near the CD4 binding site and coreceptor (CCR5) binding site, exhibited greatest potency and breadth against multiclades of HIV-1 strains. Our data therefore demonstrate that GPI-scFvs may be developed as a useful tool to identify antibodies that recognize transiently-exposed epitopes in glycoproteins spike of HIV and other enveloped viruses. GPI-scFvs derived from such antibodies could be an effective way to inhibit cell entry of HIV and other enveloped viruses.

## Methods

### Viruses and cell lines

Proviral plasmids pBru-3, pBru-Yu2, pAD8, pNL4-3 and pMj4 were obtained from the NIH AIDS Research and Reference Reagent Program (ARRRP, Germantown, MD). Infectious virus stocks of HIV-1 Bru-3 and NL4-3 (X4 tropic, subtype B), Bru-Yu2, and AD8 (R5 tropic, subtype B) and Mj4 (R5 tropic, subtype C) were produced by transfecting into 293T cells using a calcium phosphate precipitation or a Fugene-6 reagent method (Roche Diagnostics, Mannheim, Germany). Virus stocks were then propagated by infecting phytohemagglutinin (PHA)-stimulated human peripheral blood mononuclear cells (PBMCs) with these infectious clones as described before [24]. Primary HIV-1 isolates RJ407 and JS-JCD were generated by infecting PHA-stimulated human PBMCs with plasmas of two subjects in a cohort of individuals who was recruited for a study of HIV-1 fitness. Informed consent was obtained from participants in accordance with the Institutional Review Boards of the Huashan Hospital, Shanghai, China. All virus stocks (Bru-3, NL4-3, Bru-Yu2, AD8, Mj4, RJ407, and JS-JCD) were stored in aliquots at a -80°C degree freezer. The titers of the stocks were determined by end-point titration on TZM.bl cells (see below).

The packaging cell line 293T was purchased from Invitrogen Life Technologies (San Diego, CA) and maintained in complete DMEM medium [i.e. high glucose DMEM supplemented with 10% FBS, 2 mM L-glutamine, 1 mM sodium pyruvate, penicillin (100 U/ml), streptomycin (100 μg/ml)] plus G418 (500 μg/ml) (Invitrogen Life Technologies). Human CD4^+ ^T cell line CEMss was obtained from Dr. Jon Allan at the Department of Virology and Immunology, the Southwest Foundation for Biomedical Research, San Antonio, TX. TZM.bl and 69Tirev/env cells were obtained from NIH ARRRP, as contributed by J. Kappes and X. Wu [58,59] and H. Yu [44], respectively. CEMss cells and TZM.bl cells were maintained in complete DMEM. 69TiRev/Env cells were maintained in complete DMEM plus 0.2 mg/ml G418.

*Drosophila *Schneider 2 (S2) cells were purchased from Invitrogen Life Technologies and maintained in 75 cm flask (Corning) at the cell density between 0.2 to 2 × 10^6 ^cells per ml in the Express FIVE^® ^SFM medium (Invitrogen Life Technologies) supplemented with 10% FBS, 100 U/ml penicillin, 100 U/ml streptomycin and 100 mg/L L-glutamine at 28°C without additional CO_2_.

### Gene construction

To construct the scFv/IgG3 hinge/his-tag/DAF, a 143-bp DNA fragment containing a 8-bp sequence of 3'end of human IgG3 hinge region, a 123-bp sequence encoding a 6 x his tag, the C-terminal 34 amino acid residues of DAF [37] and a stop codon, and a 12-bp sequence of a *Sal I *site and additional 6 bp were linked by a recursive PCR [60]. Another DNA fragment containing a 12-bp sequence of a *Bgl II *site and additional 6 bp, a sequence encoding the signal peptide, anti-HIV-1 gp41 scFv (TG15) and 11 residues of human IgG3 hinge region, and an 8-bp sequence of the 5'end of the his tag was amplified by PCR using previously generated pLNCX-TCR-m-scFv as a template [23]. These two fragments were designed in such a way that there were 16 nucleotides overlapping between the 3'end of scFv/hinge and 5'end of his tag/DAF fragments. In addition, an *Xba I *site was inserted between the sequences of the signal peptide and the scFv and a *Xma I *site was inserted between the sequences of scFv and IgG3 hinge region to facilitate cloning process of other scFvs (see below). The whole fragment encoding the entire scFv(TG15)/hinge/his-tag/DAF was then generated by an overlapping PCR. The amplified cDNA was then ligated in a TA vector system (Invitrogen Life Technologies) for sequence analysis. cDNA containing the correct scFv(TG15)/hinge/his-tag/DAF was cloned into the *Bam HI *and *Sal I *sites of lentiviral transfer vector pRRLsin-18.PPT.hPGK.Wpre [61]. The resulting lentiviral transfer construct was designated as pRRL-scFv(TG15)/hinge/his-tag/DAF (Figure 1A).

To construct genes encoding scFvs (AB31, AB32, 4E10, X5 and 48d), overlapping primers for the recursive PCR were synthesized according to the published sequences [34,62]. The end primers of recursive PCR were designed in such a way that the *Xba I *and the *Xma I *sites were introduced at 5' and 3' ends of the scFv genes, respectively. The gene encoding scFv (AB65) was derived from the VH and VL fragments of a human monoclonal antibody generated from convalescent plasma of H5N1 avian influenza virus infected patient in our laboratory. The amplified genes encoding scFvs were ligated in a TA vector system for sequencing. cDNA containing the correct scFvs were then inserted into the *Xba I *and *Xma I *sites of pRRL-scFv(TG15)/hinge/his-tag/DAF. The resulting lentiviral transfer constructs were designated as pRRL-scFv(AB31, AB32, 4E10, X5, 48d and AB65)/hinge/his-tag/DAF, respectively (see Figure 1A and Additional File 1).

To construct a secretory form of the scFvs, a gene encoding sequence containing the signal peptide, scFv (TG15), IgG3 hinge, his-tag and a stop codon was first amplified by PCR using the pRRL-scFv(TG15)/hinge/his-tag/DAF as a template. The correct sequence was inserted into the *Bam HI *and the *Sal I *sites of pRRL-scFv(TG15)/hinge/his-tag/DAF to generate the lentiviral transfer construct pRRL-scFv(TG15)/hinge/his-tag. Then sequences encoding each of the other scFvs (AB31, AB32, X5, 4E10, 48d and AB65) were inserted into the *Xba I *and the *Xma I *sites of pRRL-scFv(TG15)/hinge/his-tag. The resulting lentiviral transfer constructs were designated as pRRL-scFv(AB31, AB32, X5, 4E10, 48d and AB65)/hinge/his-tag, respectively (Figure 1A and Additional File 1).

To generate genes encoding soluble human CD4 protein to be produced by *Drosophila *S2 cells, a gene encoding extracellular domain of the human CD4 protein with a his-tag at its C-terminus was PCR-amplified using total RNA isolated from CEMss cells as a template and ligated into the TA vector as described above. The correct gene sequence was then inserted into *Bgl II *and *BstB I *sites of an inducible S2 expression vector pMT-bip/V5-His (Invitrogen). The resulting plasmid was designated as pMT-bip-sCD4.

The gene encoding retroviral envelope 10A1 was PCR-amplified from the packaging cell line PT67 originally developed in Dusty Miller's laboratory [39]. The gene encoding the HIV-1 envelope Yu2 was PCR-amplified from proviral DNA (Bru-Yu2) (see above). The correct sequences (10A1 and Yu2) were then cloned into the *Sal I *and the *Bam HI *sites of CMV/R expression vector [38]. The resulting plasmids were designated as CMV/R-env (10A1) and CMV/R-env (Yu2), respectively.

The gene encoding human CCR5 was PCR amplified using total RNA isolated from human PBMCs as a template and ligated into the TA vector as described above. The correct gene sequence was then inserted into *Bam HI *and *Sal I *sites of a lentiviral transfer vector pRRLsin-18.PPT.hPGK.Wpre. The resulting lentiviral transfer construct was designated as pRRL-CCR5.

### Generation of recombinant lentiviral vectors

Recombinant lentiviral vectors were generated as described before [23]. Briefly, 4 × 10^6 ^293T cells were seeded onto P-100 dish in 10 ml complete DMEM. After overnight culture, cells were co-transfected with 20 μg transfer construct (one of pRRL-scFv/hinge/his-tag/DAFs or pRRL-scFv/hinge/his-tags as well as pRRL-CCR5), 10 μg packaging construct encoding HIV-1 gag/pol (pLP1), 7.5 μg plasmids encoding VSV-G envelope (pLP/VSVG) and 7.5 μg HIV-1 rev protein (pLP2) (Invitrogen) using a calcium phosphate precipitation method. Sixteen hours later, culture supernatants were removed and replaced with fresh complete DMEM plus 1 mM sodium butyrate (Sigma). Eight hours later, supernatants were again removed and replaced with fresh DMEM plus 4% FBS. After another 20 hours, the culture supernatants were harvested and concentrated by ultra-centrifugation as described before [38]. The vector pellets were resuspended in a small volume of DMEM and stored in aliquots at -80°C degree freezer. Vector titers were determined as we previously described [23]. The amount of HIV-1 gag p24 in concentrated vector stocks was determined by ELISA (see below).

### Generation of stable CEMss-CCR5 cells

To generate CEMss-CCR5 cells, 1 × 10^5 ^CEMss cells and 2 × 10^6 ^TU of recombinant lentiviral vector expressing human CCR5 were added onto 24-well tissue culture plate in the presence of 8 μg/ml of polybrene (Sigma). Twenty four hours later cells were extensively washed and cultured in complete DMEM. The expression of CCR5 was measured by FACS analyses.

### Generation of soluble CD4 (sCD4) by drosophila S2 cells

Soluble CD4 were generated according to the manufacturer's instruction. Briefly, 2 × 10^6 ^S2 cells were seeded onto each well of 6-well plate in 3 ml Express FIVE^® ^SFM medium. After overnight culture, cells were co-transfected with 19 μg pMT-sCD4 and 1 μg pCoBlast (Invitrogen life Technologies) using a calcium phosphate precipitation method. Forty eight hours later, 25 μg/ml blasticidin were added into medium to select stably cotransfected cell lines and followed by limiting dilution assay to select stable cell clones.

To produce sCD4, stable clones were induced with 5 μg/ml CdCl_2 _for 3 to 5 days. Soluble CD4 in supernatants were harvested and purified by Ni-NTA column (Invitrogen) according to manufacturer's instruction. The amount of proteins was quantified by BCA assay (Pierce) and the purity of proteins was determined by 12% SDS/PAGE followed by Coomassie blue staining.

### Generation of stable cell lines expressing secretory and GPI-scFvs

To transduce CEMss and CEMss-CCR5 cells, 1 × 10^5 ^CEMss or CEMss-CCR5 cells and 2 × 10^6 ^TU of one of the lentiviral vectors containing pRRL-scFv (X5 or AB65)/hinge/his-tag/DAF or pRRL-scFv(X5 or AB65)/hinge/his-tag were added onto 24-well tissue culture plate in the presence of 8 μg/ml of polybrene (Sigma). Twenty four hours later cells were extensively washed and cultured in complete DMEM. To transduce TZM.bl cells, 5 × 10^4 ^TMZ.bl cells per well were seeded onto 24 well plate. After overnight culture, 2 × 10^6 ^TU of one of lentiviral vectors containing pRRL-scFv (TG15, AB31, AB32, 4E10, X5, 48d or AB65)/hinge/his-tag/DAF or pRRL-scFv (TG15, AB31, AB32, 4E10, X5, 48d or AB65)/hinge/his-tag were added onto 24-well tissue culture plate in the presence of 8 μg/ml of polybrene. Twenty four hours later cells were extensively washed and cultured in complete DMEM. Expression of scFv/hinge/his-tag/DAF constructs was measured by FACS and western blot analyses and expression of scFv/hinge/his-tag was measured by western blot analysis (see below). We usually found that after a single round transduction, over 98% cells express transgenes (data not shown). After transduced cells were generated, cells were continuously cultured in complete DMEM and split every 3 or 4 days. Periodically, expression of transgenes was measured. We found that the level of transgene expression was very stable in the transduced cell lines (data not shown).

### FACS analysis

To study cell surface expression of scFv/hinge/his-tag/DAF, 2 × 10^5 ^mock- and scFv (TG15, AB31, AB32, 4E10, X5, 48d and AB65)/hinge/his-tag/DAF-transduced TZM.bl cells, mock- and scFv (X5 and AB65)/hinge/his-tag/DAF-transduced CEMss-CCR5 cells were incubated with a mouse anti-his-tag antibody (Sigma) for 45 min on ice. Cells then were washed twice with FACS buffer (PBS containing 1% BSA and 0.02% NaN_3_) and stained with PE-conjugated goat anti-mouse IgG antibody (Sigma) for another 45 min on ice. Cells then were washed twice with FACS buffer and fixed with 1% formaldehyde in 0.5 ml of FACS buffer. FACS analysis was performed on a FACScan (Becton Dickinson, Mountain View, CA).

To determine whether the expression of scFv (TG15, AB31, AB32, X5, 48d and AB65)/hinge/his-tag/DAF is truly through a GPI anchor, 2 × 10^5 ^mock- and scFv/hinge/his-tag/DAF-transduced TMZ.bl and CEMss-CCR5 cells were first incubated with or without 3 μg/ml PI-PLC (Prozyme, San Leandro, CA, USA) in DMEM containing 1% FBS at 37°C for 30 min. After the incubation, cells were washed twice to remove remaining PI-PLC and then stained with a mouse anti-his-tag antibody as described above.

### Western blot analysis

1×10^6 ^cells [TZM.bl-GPI-scFvs (AB65, TG15, AB31, AB32, X5 and 48d), TZM.bl-scFvs (AB65, TG15, AB31, AB32, X5 and 48d), CEMss-R5-GPI-scFvs(AB65 and X5) and CEMss-R5-scFvs(AB65 and X5)] were grown in the DMEM plus 1% FBS on the 12-well plate for 24 hours. Cells and supernatant were harvested respectively. Cells were lysed by lysis buffer (100 mM Tris-HCl, pH8.0, 1% NP40) in the presence of protease inhibitor cocktail (Calbiochem). Proteins in supernatant were precipitated by TCA and dissolved in the equal volume of lysis buffer that to lysed cell pellet. Samples were detected by mouse anti-His tag antibody (Sigma) and mouse anti human beta-tubulin antibody as a reference control.

### Generation of pseudotypes of HIV-1 vector and a single-cycle infectivity assay

To generate pseudotypes with the HIV-1 vector, 4 × 10^6 ^293T packaging cells were co-transfected with 10 μg of HIV-1-luciferase transfer vector [38] and 10 μg of DNA plasmid encoding one of HIV-1 envelopes (Q168, AD8, ADA, JR-FL, Yu2, HxBc2, consensus B, Mj4, CN054, CNE8, CNE11 or CNE17) or control retroviral envelope 10A1 using a calcium phosphate precipitation method (see above). DNA plasmids encoding HIV-1 envelopes Q168, AD8, ADA, JR-FL, HxBc2 and Mj4 were obtained from ARRRP. DNA plasmid encoding consensus B was a generous gift of Dr. B.H. Hahn at the University of Alabama. DNA plasmids encoding CNE8, CNE11 or CNE17 were obtained from Dr. Linqi Zhang at the Tsinghua University. HIV-1 envelope Q168 is derived from a R5 tropic clade A virus [63]. HIV-1 envelopes AD8, ADA, JR-FL, Yu2 are derived from R5 tropic clade B viruses [64-67]. HIV-1 envelope HxBc2 is derived from a X4 tropic clade B virus [68]. HIV-1 envelope consensus B is an artificial envelope originally generated in Dr. Hahn's laboratory [69]. HIV-1 envelopes Mj4 is derived from R5 tropic clade C viruses [70]. HIV-1 envelope CNE8 is derived from a R5 tropic clade CRF01_AE virus. HIV-1 envelope CNE11 is derived from a R5 tropic clade B' virus. HIV-1 envelope CNE17 is derived from a R5 tropic clade CRF07_B'C virus (Zhang et al. unpublished data). The pseudotype-containing supernatants were harvested and stored in aliquots at -80°C degree freezer. The amount of HIV-1 p24 in collected supernatants was measured by ELISA.

In a single-cycle assay to measure the infectivity of pseudotypes, 10,000 Mock-, scFv/hinge/his-tag-, scFv/hinge/his-tag/DAF-transduced TZM.bl cells were transduced with HIV-1 pseudotype-containing supernatants equivalent to relative luciferase activity 300,000 to 1,000,000 overnight. Cells were then washed twice with PBS and cultured in complete DMEM medium for 2 days. Cells were then washed once with PBS and lysed in 100 μl of lysis buffer. Luciferase activity in 50 μl of cell suspensions was measured by a BrightGlo Luciferase assay according to the manufacturer's instruction (Promega).

### HIV-1 infection and luciferase and p24 assays

Mock-, scFv/hinge/his-tag-, scFv/hinge/his-tag/DAF-transduced TMZ.bl cells at 5,000 cells per well were seeded onto 96-well plate. After the overnight culture, cells were infected with HIV-1 strains Bru-3, Bru-YU2, AD8, Mj4, RJ407 and JS-JCD (100 to 200 TCID_50_) in a final volume of 0.5 ml overnight. Cells were then washed three times with HBSS and resuspended in 2 ml of the complete DMEM medium and incubated at 37°C for 2 days. Infectivity of HIV-1 was determined by a BrightGlo Luciferase assay (see above).

To test the effect of sCD4 on anti-HIV potency of secretory and GPI-anchored scFvs (X5), parental TZM.bl cells, TZM.bl-scFv (X5) and TZM.bl-GPI-scFv (X5) (5,000 cells per well) were seeded onto 96-well plate and incubated overnight. 400 TCID_50 _and 4,000 TCID_50 _of HIV-1 Bru-3 and Bru-Yu2 were first mixed with or without pre-determined 0.3 μg/ml of sCD4 at 37°C for 15 minutes. Viruses with or without sCD4 were then added onto parental TZM.bl cells, TZM.bl-scFv (X5) and TZM.bl-GPI-scFv (X5) cells. After 2 days, infectivity of HIV-1 was determined by BrightGlo Luciferase assay (see above).

### Long term culture of HIV-1 infected cells

1×10^6 ^CEMss-CCR5 cells transduced with GPI-scFvs (X5 or AB65) and secretory scFvs (X5 or AB65) were infected with HIV-1 strains Bru-3 and Bru-Yu2 (200 TCID_50_) in a final volume of 0.5 ml overnight. Cells were then extensively washed with HBSS, resuspended in 6 ml of complete DMEM and cultured for 3 to 15 weeks. Every 3 days, 4.5 ml of cell suspensions were harvested and replaced with the fresh medium. The supernatants were then collected. HIV-1 p24 in the supernatants were measured by ELISA (Beckman Coulter) according to the manufacturer's instruction.

### Cell-cell fusion experiment

69TiRev/Env cells (1×10^5 ^per well) with or without 2 μg/ml tetracycline treatment were grown on 12-well plate at 37°C overnight. CEMss-GPI-scFvs (AB65 or X5) (2×10^5^) were then added onto 69TiRev/Env cells. Cell-cell fusion was monitored under the light microscopy at various time intervals. When fusion process reached peak at 20 hours, the results were recorded by CCD digital camera (Leica DM IRB).

### Ex vivo generation of human DC

Peripheral blood was obtained from healthy donors at the Gulf Coast Regional Blood Center, Houston, Texas. Peripheral blood mononuclear cells (PBMCs) were isolated using Ficoll density gradient centrifugation as described before [71]. To isolate CD14^+ ^monocytes, the anti-CD14 microbeads and miniMACS system were used according to manufacturer's instructions (Miltenyi Biotech, Auburn, CA). Briefly, 10^8 ^PBMCs were resuspended in 800 μl of binding buffer (PBS containing 0.5% BSA and 2 mM EDTA). 200 μl of MACS CD14 microbeads were added, mixed and incubated at 4°C for 15 minutes. The cell-bead mixture was then washed once with binding buffer and resuspended in 1 ml of fresh binding buffer. CD14^+ ^cells were positively selected using an MS+/RS^+ ^column tip. The isolated cells were washed once with complete RPMI and cultured in the complete RPMI supplemented with GM-CSF (1000 U/ml) and IL-4 (1000 U/ml) (R & D System, Minneapolis, MN) for 7 days to generate immature DCs as described before [72]. The expression of DC-SIGN on the cell surface of immature DCs was measured by anti-DC-SIGN antibody staining followed by FACS analysis as described before [23].

### Capture and transfer assay with human DCs

DCs (10^5 ^per well) were seeded in triplicates in a 96 well U-bottom plate and incubated with 100 TCID_50 _of NL4-3 in a total volume of 200 μl at 37°C and 5% CO_2 _for 4 hours. Cells were then washed three times with PBS to remove cell-free virus and resuspended in 2 ml of complete RPMI into wells of 24-well plate with or without adding 1.5 × 10^5 ^CEMss-GPI-scFvs (AB65 and X5) cells. Cells were continued to be cultured for 14 days. Every 3 or 4 days, medium was removed from each well and replaced with fresh complete medium. HIV-1 p24 in the supernatants was measured by ELISA as described above.

### Immunofluorescent staining and confocal analysis

Parental and TZM.bl-GPI-scFvs (AB65 and X5) cells were seeded (5,000 cells per well) onto the Lab-Tek chamber slide (Nalge Nunc International, Rochester, NY) and incubated at 37°C 5% CO_2 _for 2 days. Cells were then washed twice with 500 μl PBS and fixed by fixation buffer (4% formaldehyde in PBS plus 1%BSA) for 15 min. Cells were washed twice with 500 μl PBS and blocked with blockage buffer (5% goat serum in PBS plus 1%BSA) for 1 hour. Cells were stained with Alexa 555 conjugated CtxB (Invitrogen Life Technologies) at 4°C for 45 min. After washed 3 times with PBS, cells stained with mouse anti-his-tag antibody (Sigma) at 4°C for 45 min, then stained with Alexa 488-conjugated goat anti-mouse IgG antibody (Invitrogen) at 4°C. After cells were washed 3 times with PBS, cells were stained with 4',6-diamidino-2-phenylindole (DAPI) in permeabilization buffer (blockage buffer plus 0.5% saponin) for 5 min. The slides were mounted before being analyzed under confocal fluorescent microscope (Zeiss Model LSM 510).

## Competing interests

The authors declare that they have no competing interests.

## Authors' contributions

MW designed and performed the experiments and participated in writing the manuscript. RA contributed to the experiments in human DC transferred and captured HIV part. QHW contributed to GPI anchored scFv (TG15) gene construction. LHL contributed to protein expression and data analysis. JTK participated in the design of the study and helped to draft the manuscript. PZ designed the experiments, wrote and completed the manuscript. All authors read and approved the final manuscript.

## Supplementary Material

Additional file 1 **Supplementary Figure 1. Expression of GPI-scFvs (AB65 and X5) in transduced TZM.bl cells**. ***a. ***Schematic diagram of the lentiviral vectors pRRL-scFv(4E10)/hinge/his-tag/DAF and pRRL-scFv(4E10)/hinge/his-tag. ***b. ***FACS analysis of cell surface expression of scFv/hinge/histag/DAF in mock-, scFvs (AB65, X5 and 4E10)/hinge/histag/DAF. Click here for file

Additional file 2 **Supplementary Figure 2. Effect of transgenes on cell surface of expression of HIV-1 receptor and co-receptors in TZM.bl cells**. ***a. ***Cell surface expression of CD4, CCR5 and CXCR4 of parental TZM.bl cells (mock) and TZM.bl cells transduced with lentiviral vectors expressing GPI-scFvs (AB31, AB32, TG15, 48d, X5 and AB65). ***b. ***Cell surface expression of CD4, CCR5 and CXCR4 of parental TZM.bl cells (mock) and TZM.bl cells transduced with lentiviral vectors expressing secretory scFvs (AB31, AB32, TG15, 48d, X5 and AB65). ***c. ***Side by side comparison of cell surface expression of CD4, CCR5 and CXCR4 of parental TZM.bl cells (mock) and TZM.bl cells transduced with lentiviral vectors expressing GPI-scFvs (AB65, X5 and 4E10) or secretory scFvs (AB65, X5 and 4E10). Click here for file

Additional file 3 **Supplementary Figure 3. Expression of soluble CD4**. Coomassie blue staining of purified soluble CD4 by transfected drosophila S2 cells and separated by 12% SDS/PAGE. Click here for file

Additional file 4 **Supplementary Figure 4. Expression of secretory and GPI-scFvs (AB65 and X5) in transduced CEMss-CCR5 cells**. ***a. ***Western blot analysis of expression of scFvs (X5 and AB65) in CEMss-CCR5 cells transduced with lentiviral vectors pRRL-scFv/hinge/his-tag/DAF (AB65 and X5) and pRRL-scFv/hinge/his-tag (AB65 and X5). GPI-scFv: GPI-anchored scFv; Sec scFv: secretory scFv; anti-his: anti-his-tag antibody. ***b. ***FACS analysis of cell surface expression of scFv/hinge/histag/DAF in mock-, scFvs (X5 and AB65)/hinge/histag/DAF- or m-scFv(TG15)-transduced CEMss-CCR5 cells with or without PI-PLC treatment. ***c. ***Cell surface expression of CD4, CCR5 and CXCR4 of parental CEMss-CCR5 cells (mock) and CEMss-CCR5 cells transduced with lentiviral vectors expressing GPI-scFvs (X5 and AB65) and secretory scFvs (X5 and AB65). Click here for file
