# Supplementary figures and images for: GPI-anchored single chain Fv - an effective way to capture transiently-exposed neutralization epitopes on HIV-1 envelope spike
Source: Retrovirology. 2010 Oct 6;7:79. doi: 10.1186/1742-4690-7-79 (PMC2959034; doi:10.1186/1742-4690-7-79)

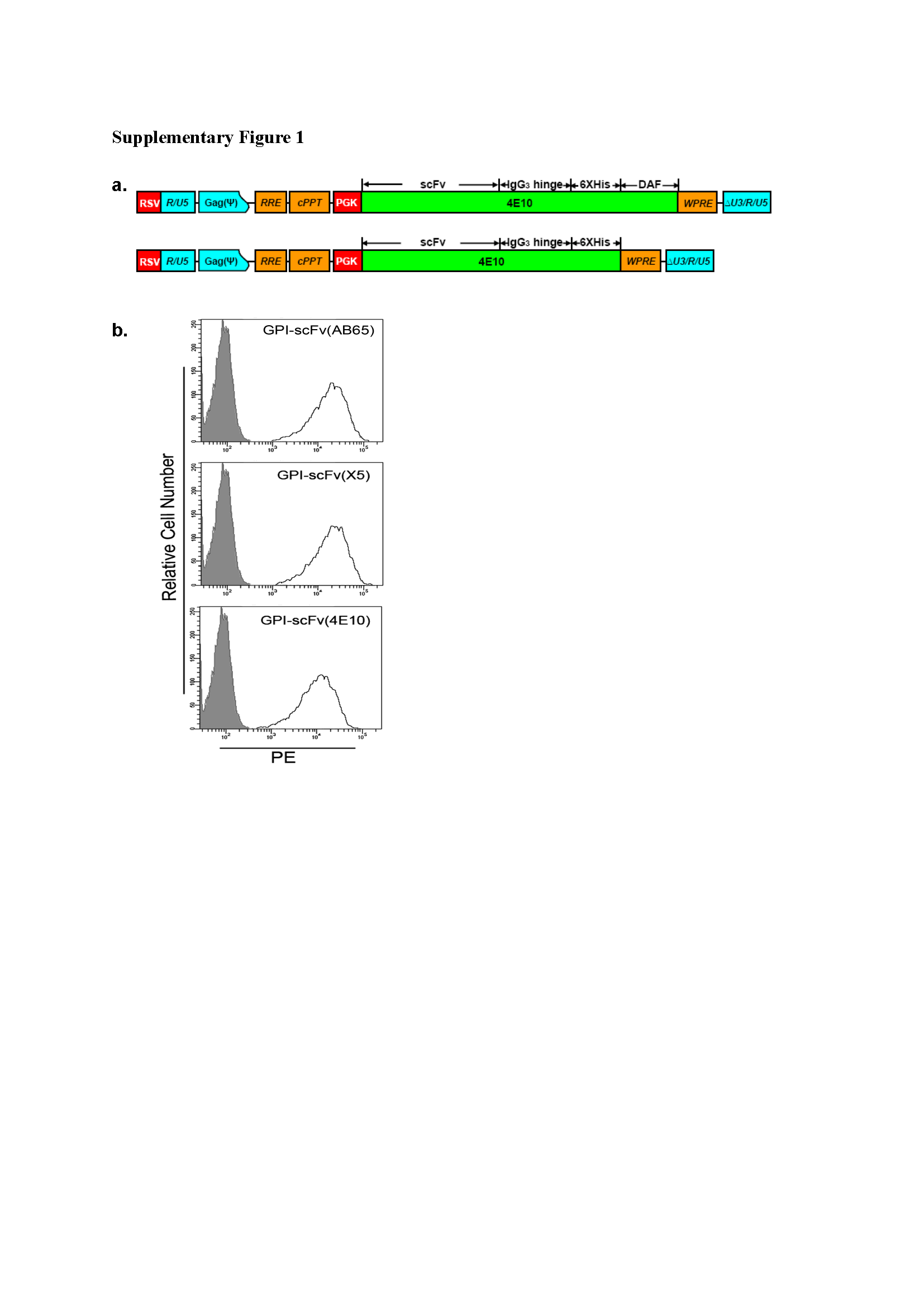

Supplement: Additional file 1 — Supplementary Figure 1. Expression of GPI-scFvs (AB65 and X5) in transduced TZM.bl cells. a. Schematic diagram of the lentiviral vectors pRRL-scFv(4E10)/hinge/his-tag/DAF and pRRL-scFv(4E10)/hinge/his-tag. b. FACS analysis of cell surface expression of scFv/hinge/histag/DAF in mock-, scFvs (AB65, X5 and 4E10)/hinge/histag/DAF. [file 1742-4690-7-79-S1.TIFF]

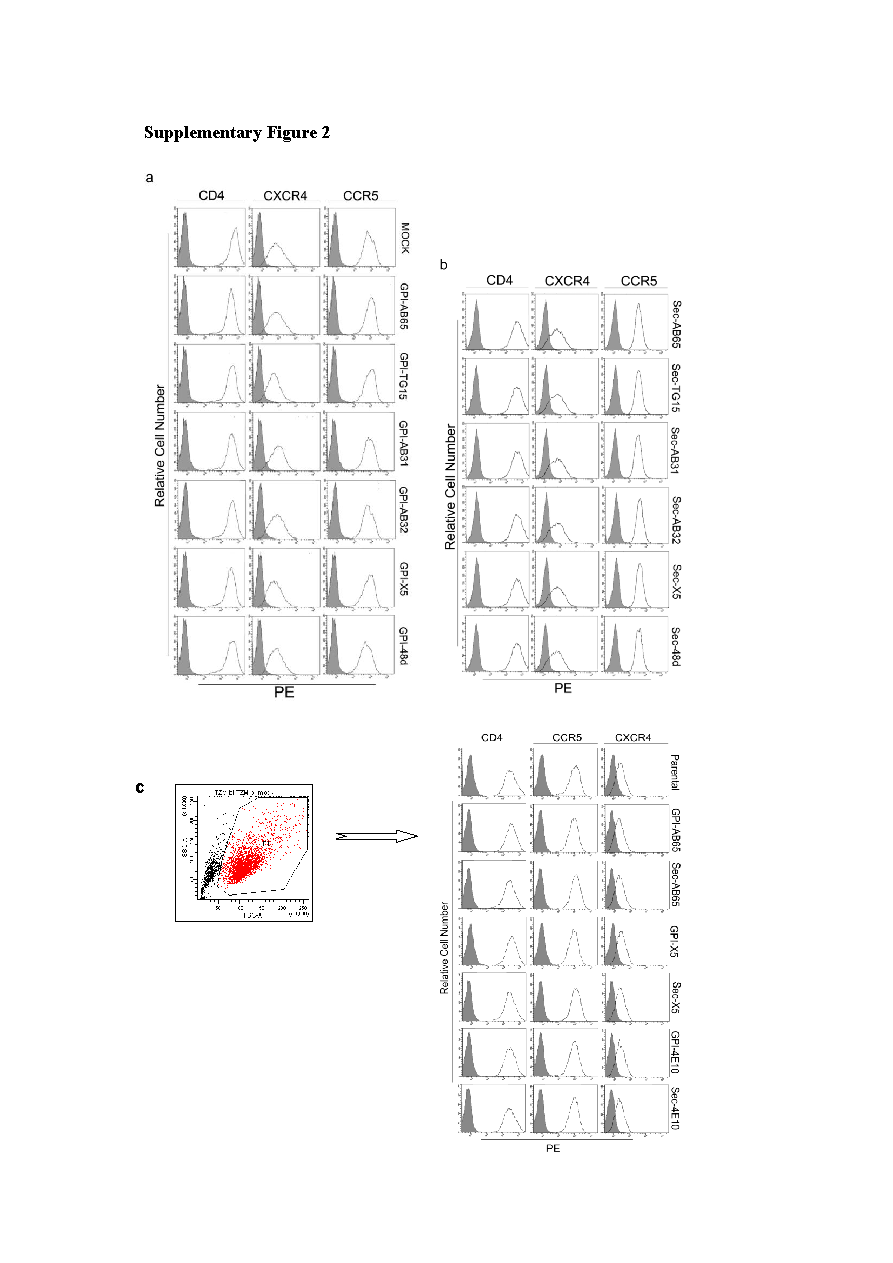

Supplement: Additional file 2 — Supplementary Figure 2. Effect of transgenes on cell surface of expression of HIV-1 receptor and co-receptors in TZM.bl cells. a. Cell surface expression of CD4, CCR5 and CXCR4 of parental TZM.bl cells (mock) and TZM.bl cells transduced with lentiviral vectors expressing GPI-scFvs (AB31, AB32, TG15, 48d, X5 and AB65). b. Cell surface expression of CD4, CCR5 and CXCR4 of parental TZM.bl cells (mock) and TZM.bl cells transduced with lentiviral vectors expressing secretory scFvs (AB31, AB32, TG15, 48d, X5 and AB65). c. Side by side comparison of cell surface expression of CD4, CCR5 and CXCR4 of parental TZM.bl cells (mock) and TZM.bl cells transduced with lentiviral vectors expressing GPI-scFvs (AB65, X5 and 4E10) or secretory scFvs (AB65, X5 and 4E10). [file 1742-4690-7-79-S2.TIFF]

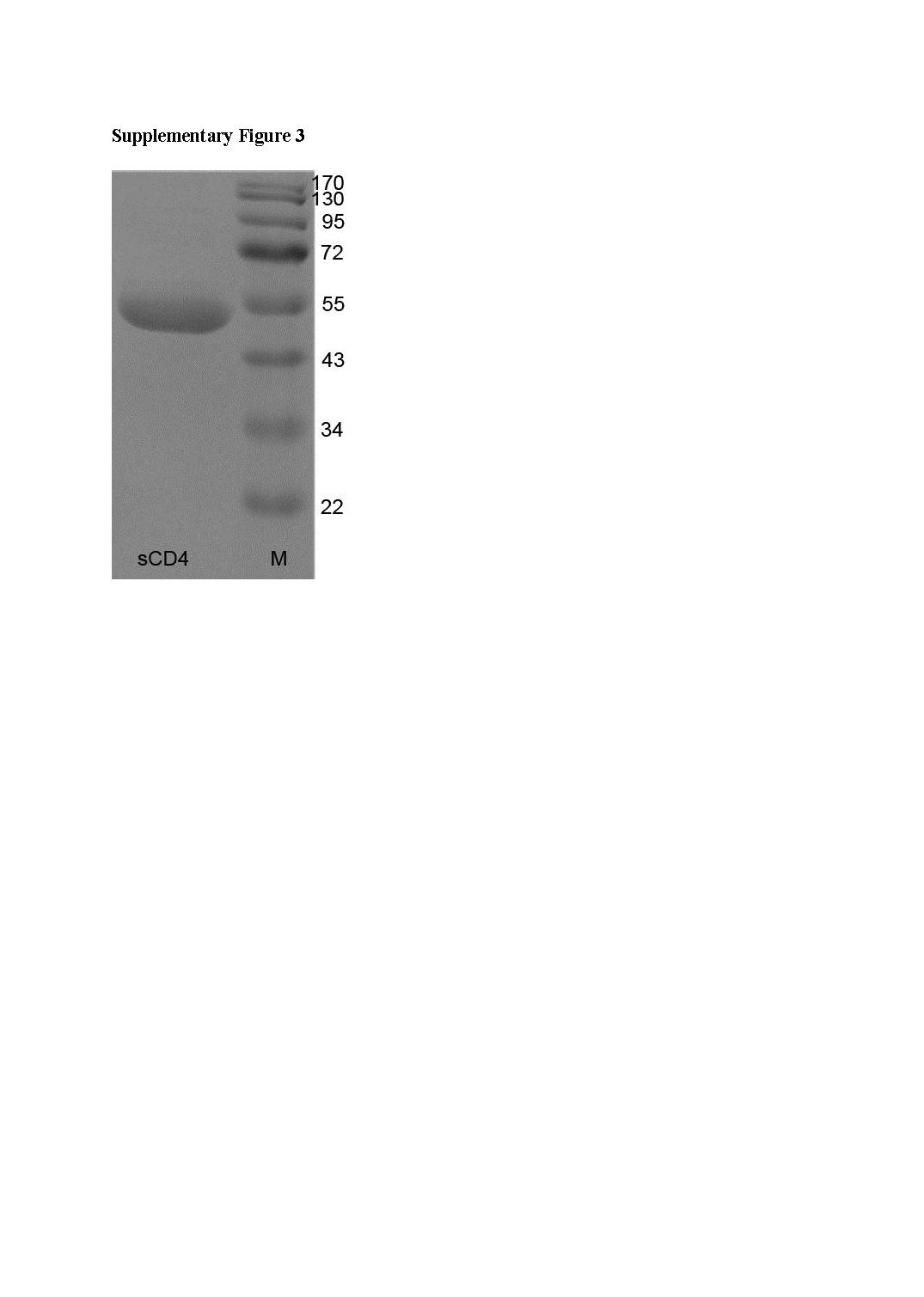

Supplement: Additional file 3 — Supplementary Figure 3. Expression of soluble CD4. Coomassie blue staining of purified soluble CD4 by transfected drosophila S2 cells and separated by 12% SDS/PAGE. [file 1742-4690-7-79-S3.TIFF]

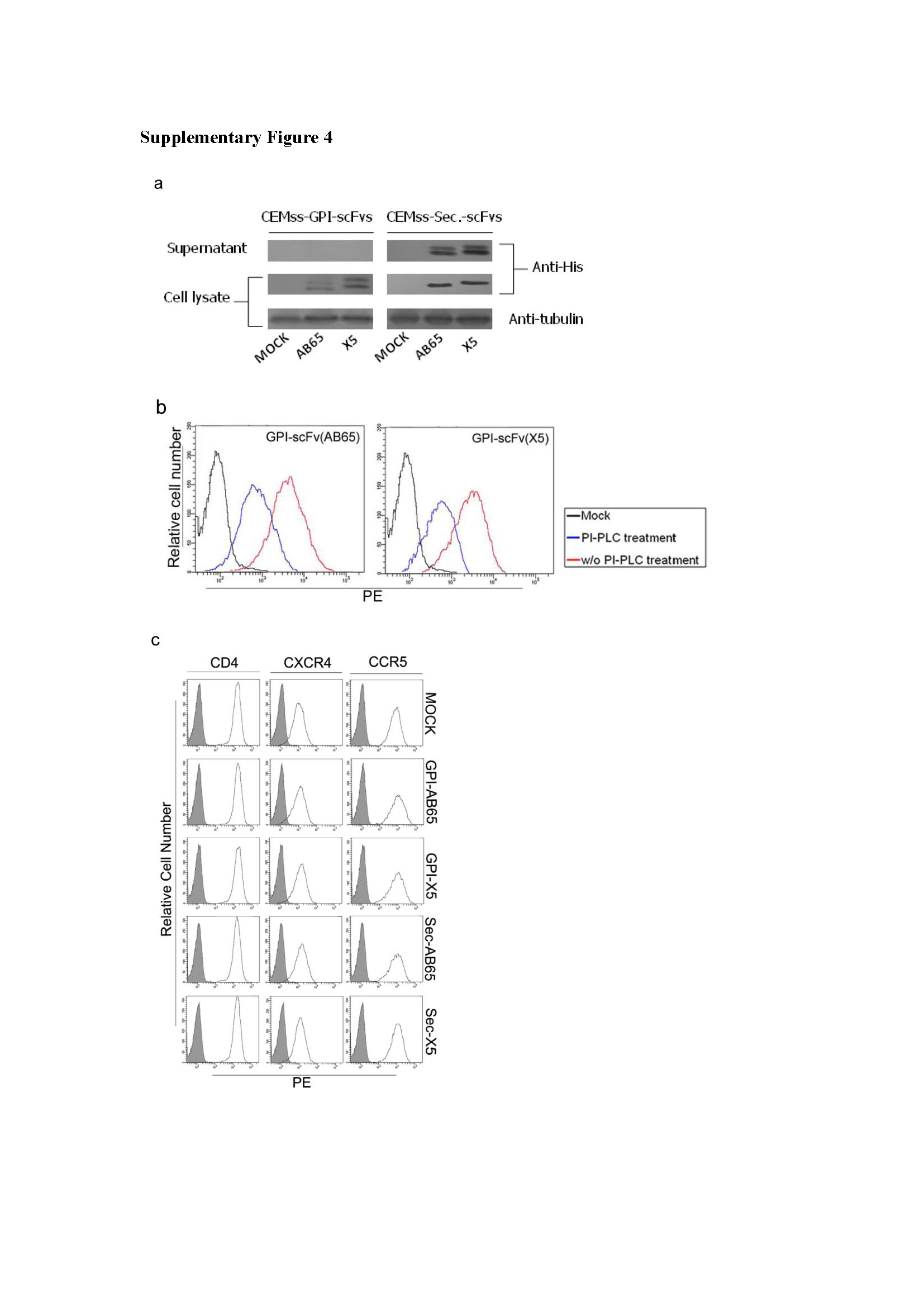

Supplement: Additional file 4 — Supplementary Figure 4. Expression of secretory and GPI-scFvs (AB65 and X5) in transduced CEMss-CCR5 cells. a. Western blot analysis of expression of scFvs (X5 and AB65) in CEMss-CCR5 cells transduced with lentiviral vectors pRRL-scFv/hinge/his-tag/DAF (AB65 and X5) and pRRL-scFv/hinge/his-tag (AB65 and X5). GPI-scFv: GPI-anchored scFv; Sec scFv: secretory scFv; anti-his: anti-his-tag antibody. b. FACS analysis of cell surface expression of scFv/hinge/histag/DAF in mock-, scFvs (X5 and AB65)/hinge/histag/DAF- or m-scFv(TG15)-transduced CEMss-CCR5 cells with or without PI-PLC treatment. c. Cell surface expression of CD4, CCR5 and CXCR4 of parental CEMss-CCR5 cells (mock) and CEMss-CCR5 cells transduced with lentiviral vectors expressing GPI-scFvs (X5 and AB65) and secretory scFvs (X5 and AB65). [file 1742-4690-7-79-S4.TIFF]
